# *IRF4* rearrangement may predict favorable prognosis in children and young adults with primary head and neck large B‐cell lymphoma

**DOI:** 10.1002/cam4.5828

**Published:** 2023-04-20

**Authors:** Xiang‐Nan Jiang, Fang Yu, Tian Xue, Qing‐Xin Xia, Qian‐Ming Bai, Bao‐Hua Yu, Ruo‐Hong Shui, Xiao‐Yan Zhou, Xiong‐Zeng Zhu, Jun‐Ning Cao, Xiao‐Nan Hong, Xiao‐Qiu Li

**Affiliations:** ^1^ Department of Pathology Fudan University Shanghai Cancer Center Shanghai China; ^2^ Department of Oncology Shanghai Medical College, Fudan University Shanghai China; ^3^ Institute of Pathology Fudan University Shanghai China; ^4^ Department of Pathology The First Affiliated Hospital of Zhejiang University Hangzhou China; ^5^ Department of Pathology Affiliated Cancer Hospital of Zhengzhou University Zhengzhou China; ^6^ Department of Medical Oncology Fudan University Shanghai Cancer Center Shanghai China

**Keywords:** children and young adults, head and neck, *IRF4* rearrangement, large B‐cell lymphoma, therapeutic strategy

## Abstract

**Purpose:**

Large B‐cell lymphoma with *IRF4* rearrangement (LBCL, *IRF4*+) has been recently recognized as a specific entity that is frequently associated with young age and favorable prognosis. However, whether the good outcome of the disease is due to *IRF4*+ or other factors remains obscure. We thus analyzed 100 young patients with primary head and neck LBCL to see the clinicopathologic correlates of *IRF4*+.

**Methods:**

The histopathology, immunophenotype, *IRF4* status of the tumors, and clinical data were reviewed.

**Results:**

Twenty‐one tumors were diagnosed as LBCL, *IRF4*+, which were more frequently associated with a follicular growth pattern, medium‐sized blastoid cytology, germinal center B‐cell‐like, and CD5+ phenotype, compared with IRF4− ones. While most of the patients received chemotherapy with or without radiation, eight *IRF4*+ patients received mere surgical resection of the tumor and exhibited excellent outcome. *IRF4*+ cases featured a significantly higher complete remission rate, and better survivals compared with *IRF4*− ones. Multivariate analysis confirmed *IRF4*+ correlates with a better survival.

**Conclusion:**

Our work confirmed the unique clinicopathologic features of LBCL, *IRF4*+, and disclosed for the first time the independent favorable prognostic impact of *IRF4*+. These findings may further unravel the heterogeneity of LBCL occurring in youth, and aid in risk stratification and tailoring the therapeutic strategy.

## Graphical abstract

The rearrangement of *IRF4* gene not only defines a group of LBCL with distinct clinicopathologic features, but also is of practical implications for prognosis prediction and therapeutic strategy decision.

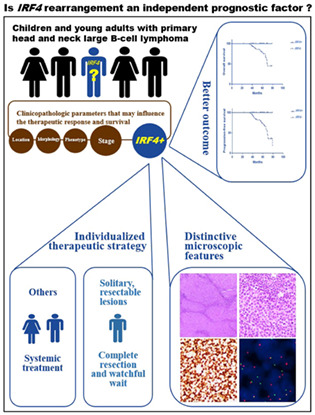

## 1 INTRODUCTION

Large B‐cell lymphoma with *IRF4* rearrangement (LBCL, *IRF4*+) was recently recognized as a specific entity characterized genetically by *IRF4* translocation (mostly with *IG* genes),^1^, ^2^ and the disease was introduced by the revised 4th edition of the World Health Organization (WHO) Classification of Hematopoietic and Lymphoid Neoplasms as a new provisional entity.^3^ This type of lymphoma affects more frequently children under 18 years of age, typically with a limited stage involving the head and neck region, such as the Waldeyer ring, and is characterized by a favorable prognosis.^4^

In 2011, the translocations of *IGH*, *IGL*, and *IGK* with *IRF4* were identified by Salaverria et al. in 20 of 427 (5%) LBCL patients. Those lymphomas were overwhelmingly of the germinal center B‐cell (GCB) subtype, and featured high‐level expression of IRF4/MUM1 protein, but lacked the t (14;18)/*BCL2* gene translocation. It was found such lymphomas possessed a distinctive gene expression profile.^1^ Further work with targeted gene sequencing and copy number array revealed additional aberrations, including frequent *IRF4* and NF*‐k*B‐related gene mutations, overexpression of the downstream genes of the NF*‐k*B pathway, losses of 17p13 and gains of chromosome 7, 11q12.3‐q25.^5^ LBCL, *IRF4*+ hence represents a previously unrecognized subset of B‐cell lymphomas with distinctive clinicopathologic characteristics and molecular alterations.

Most pediatric germinal center‐derived B‐cell non‐Hodgkin lymphomas, such as follicular lymphoma or diffuse LBCL (DLBCL), feature a relatively favorable outcome. Given the facts that most LBCL, *IRF4*+ patients are children and young adults with a GCB‐derived tumor, and the probability of *IRF4* rearrangement has been reported to decrease significantly with aging,^1^, ^6^ one may wonder whether the better outcome of LBCL, *IRF4*+ is attributable or related to the young age or the molecular changes such as *IRF4* rearrangement. In other words, whether *IRF4* rearrangement is an independent prognostic factor of LBCL in children and young adults remains unclear.

We thus conduct the current retrospective case–control study to evaluate the clinicopathologic correlates of *IRF4* rearrangement in young patients with a primary head and neck LBCL.

## 2 MATERIALS AND METHODS

### 2.1 Patients

Patients with DLBCL diagnosed between 2015 and 2020 were selected from the files of the Department of Pathology, Fudan University Shanghai Cancer Center. All of the selected cases fulfilled the following criteria: (1) patients with a Stage I–II disease of primary DLBCL of the head and neck region at the age ≤35 years, (2) transformed DLBCL from other low‐grade B‐cell lymphomas were excluded. Altogether 106 cases were collected, among which 100 with follow‐up information and available materials were employed for pathologic review. Clinical data, including sex, age, Ann Arbor stage, B symptoms, treatment, and the outcome were collected either from medical records or by telephone inquiries. The use of human tissue samples was approved by the Ethics Committee at Fudan University Shanghai Cancer Center.

### 2.2 Histological review

Formalin‐fixed, paraffin‐embedded (FFPE) specimens were used for the morphological and ancillary studies. Routine hematoxylin and eosin‐stained sections were reviewed by three of the authors (XNJ, TX, and XQL) and diagnosis was rendered according to the updated WHO classification.^3^ Consensus was reached on uncertain cases after discussion.

### 2.3 Immunohistochemistry and fluorescence in situ hybridization (FISH)

Immunohistochemical and FISH analyses were performed by using standard protocols. FFPE sections were stained with primary antibodies against CD5 (sp19, Roche), CD10 (56C6; DAKO), BCL6 (PG‐B6P; DAKO), BCL2 (124; DAKO), MYC (Y69, Epitomics), and IRF4/MUM1 (MUM1P; DAKO) on a BenchMark XT automated immunostainer (Ventana Medical Systems). A positive result was concluded if ≥30% of tumor cells were stained with antibodies against CD10, BCL6, and MUM1. GCB and non‐GCB subtypes of DLBCL were determined by the Hans algorithm.^7^ High‐level expression of BCL2 or MYC was defined, respectively, in that >50% and >40% of tumor cells were positive. Detection of breakpoints affecting the *BCL2*, *MYC* (LSI dual‐color break‐apart probes; Vysis‐Abbott), and *IRF4* (LSI dual‐color break‐apart probes; Zytovision) was performed by using FISH. A positive result was concluded when ≥15% of tumor cells demonstrating split signals. Epstein–Barr virus (EBV)‐encoded small RNA (EBER) was detected by in situ hybridization.

### 2.4 Statistics

Overall survival (OS) was calculated from the date of diagnosis to the date of death or the last follow‐up. Progression‐free survival (PFS) was calculated from the date of diagnosis to the date of disease progression, relapse, death from any cause, or the last follow‐up. The survival rates of DLBCL patients were estimated by the life table method. OS and PFS curves were generated by using the Kaplan–Meier method and the log‐rank test. Multivariate analyses were performed using a logistic regression model for response and Cox regression models for survival. All variables with a value of *p* < 0.1 in univariate analysis were submitted for multivariate model analysis. The results were considered statistically significant if a *p* value <0.05. Statistical analysis was carried out by using GraphPad Prism (8.0, GraphPad Software).

## 3 RESULTS

### 3.1 Patient characteristics and pathologic findings

The clinical and pathologic characteristics of the 100 young patients with a Stage I–II disease of primary DLBCL of the head and neck region are summarized in Table 1. *IRF4* rearrangement was detected in 21 patients. The median age of *IRF4*+ cases was 17 years, with a male to female ratio of 2:1. The most common site of involvement was cervical lymph nodes (61.9%), followed by Waldeyer ring/tonsils (38.1%). All of the *IRF4*+ cases featured an IPI score of 0–2, and all except one case were demonstrated to have Stage I disease. No obvious differences in the distribution of age, sex, tumor location, staging, or IPI score were observed between the *IRF4*+ cases and those lacking *IRF4* rearrangement (*IRF4*−).

**TABLE 1:** Clinicopathologic characteristics of 100 young patients with primary head and neck large B‐cell lymphoma.

	*IRF4*− (*n* = 79)	*IRF4+* (*n* = 21)	*p* value
Median age (range) (years)	29 (17–35)	17 (4–35)	0.4730
Gender, *n* (%)			
Male	43 (54.4)	14 (66.7)	0.3360
Female	36 (45.6)	7 (33.3)	
Location, *n* (%)			
Lymph nodes	47 (59.5)	13 (61.9)	> 0.1000
Waldeyer ring	30 (38)	8 (38.1)	> 0.1000
Nodes and Waldeyer ring	2 (2.5)	0 (0)	0.4390
Ann Arbor stage, *n* (%)			
I	62 (78.5)	20 (95.2)	0.0650
II	17 (21.5)	1 (4.8)	
IPI score, *n* (%)			
0–2	75 (95)	21 (100)	0.5810
3–4	4 (5)	0 (0)	
Histopathology			
Presence of a follicular growth pattern, *n* (%)	9 (11.4)	9 (42.9)	0.0020
Conspicuous starry sky pattern	9 (11.4)	1 (4.7)	0.1260
Cytology			
CB/IB	58 (73.4)	0 (0)	< 0.0001
CB/CC	21 (26.6)	8 (38.1)	0.1308
CB/MB	0 (0)	13 (61.9)	< 0.0001
Immunophenotype, *n* (%)			
CD10+	40 (50.6)	20 (95.2)	0.0001
BCL6+	78 (98.7)	21 (100)	> 0.1000
MUM1+	35 (44.3)	21 (100)	< 0.0001
GCB subtype	47 (59.5)	20 (95.2)	0.0010
Non‐GCB subtype	32 (40.5)	1 (4.8)	
BCL2+	20/76 (26.3)	6/20 (30)	0.0603
MYC+	24/76 (31.6)	7/20 (35)	0.0594
CD5+	1/77 (1.3)	5/21 (23.8)	0.0010
Genetics			
*BCL2* rearrangement	2/34 (5.8)	0/14 (0)	< 0.0010
*MYC* rearrangement	2/34 (5.8)	0/14 (0)	< 0.0010
EBER+	0/52 (0)	0/17 (0)	> 0.1000
Treatment			
S	4 (5.1)	8 (38)	< 0.0001
C	56 (70.9)	4 (19)	< 0.0001
S + C	10 (12.6)	8 (38)	0.1370
C + ISRT	3 (3.8)	1 (5)	0.5010
S + C + ISRT	6 (7.6)	0 (0)	> 0.1000

Abbreviations: C, chemotherapy; CB, centroblast; GCB, germinal center B‐cell‐like; IB, immunoblast; IPI, International Prognostic Index; ISRT, involved site radiation therapy; MB, medium‐sized blastoid cell; S, surgical excision.

**FIGURE 1:**
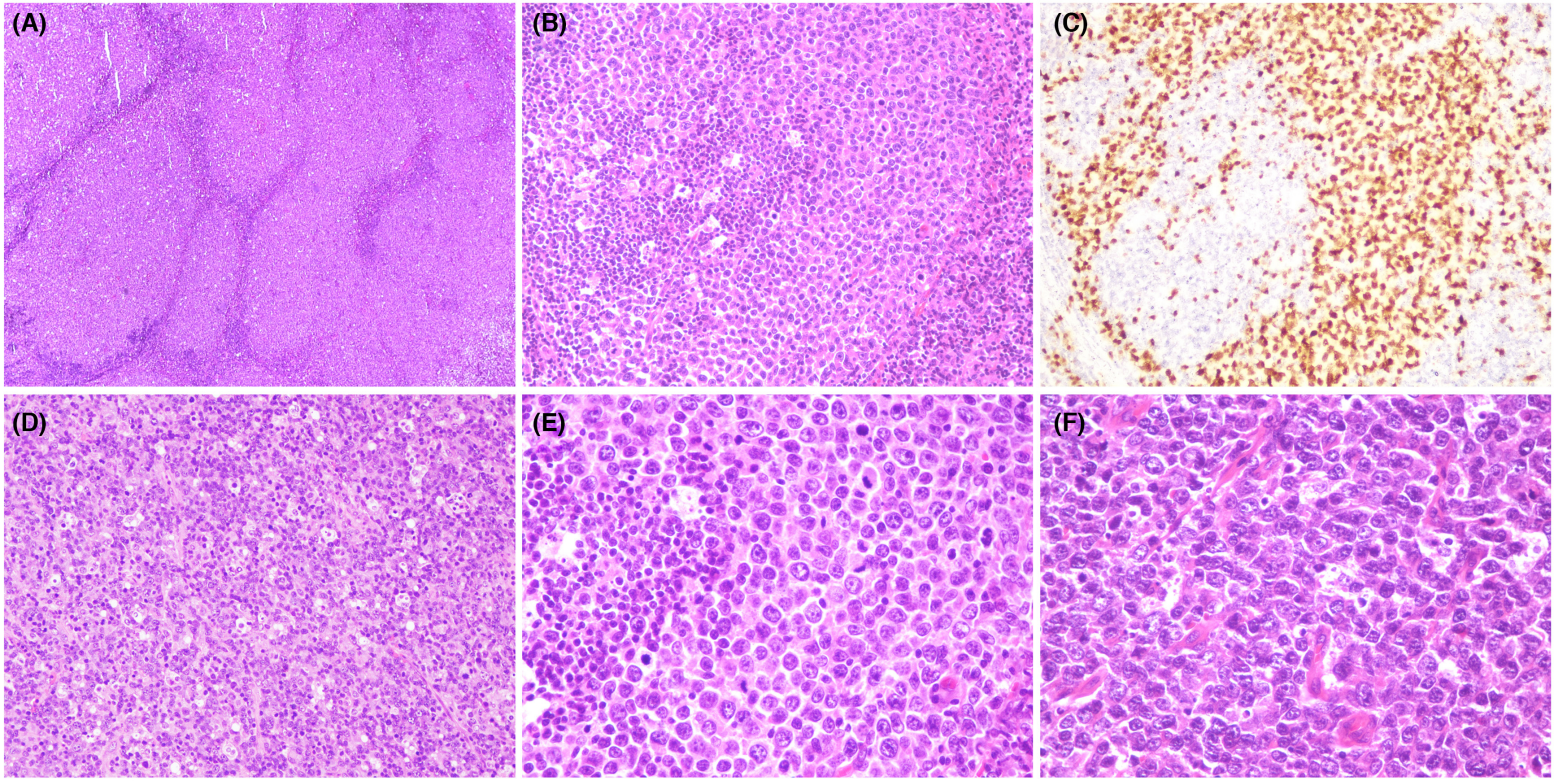
Histologically, large B‐cell lymphoma (LBCL), *IRF4*+ cases are often characterized by a follicular and diffuse lymphoid proliferation (A). Follicular colonization, that is, replacement of the peripheral outer zone of some reactive germinal centers, is evident in this case (B), which can be highlighted by IRF4/MUM1 immunostaining (C). Conspicuous starry sky pattern reminiscent of Burkitt lymphoma is occasionally present (D). Cytologically, LBCL, *IRF4*+ cases consist predominantly of monotonous medium‐sized blastoid cells (E), in contrast to the mixed cell population comprising immunoblasts, centroblasts to centrocytes noted in LBCL, *IRF4*− cases (F).

Histologically, the vast majority of the cases displayed changes compatible with a diagnosis of DLBCL. Of note, nine cases each of both cohorts displayed a concurrent follicular component, that is, follicular LBCL, in addition to the diffuse neoplastic infiltrate. Compared with *IRF4*− cases, *IRF4*+ tumors exhibited more frequently a follicular growth pattern (42.9% vs. 11.4%) (*p* = 0.0020). In the current study, we defined a follicular pattern by the presence of more than one low‐power field (2.4 mm^2^) comprising compact, or sometimes, confluent neoplastic follicles (Figure 1A). Follicular colonization, typically, replacement of the peripheral outer zone of germinal centers with lymphoma cells was seen in two cases (Figure 1B,C). Scattered tingible body macrophages were present, at least focally, in 17 cases with *IRF4*+ tumors, with a conspicuous starry sky pattern reminiscent of Burkitt lymphoma being noted in only one case (Figure 1D). Cytologically, 61.9% of all *IRF4*+ tumors were composed predominantly of atypical centroblasts to medium‐sized blastoid cells (usually with less distinct nucleoli) (Figure 1E), with the remaining ones being composed predominantly of centroblasts and centrocytes. In contrast, *IRF4*− tumors were basically composed of mixed cell population comprising immunoblasts to centrocytes or centroblasts and centrocytes (Figure 1F) (*p* < 0.0001).

**FIGURE 2:**
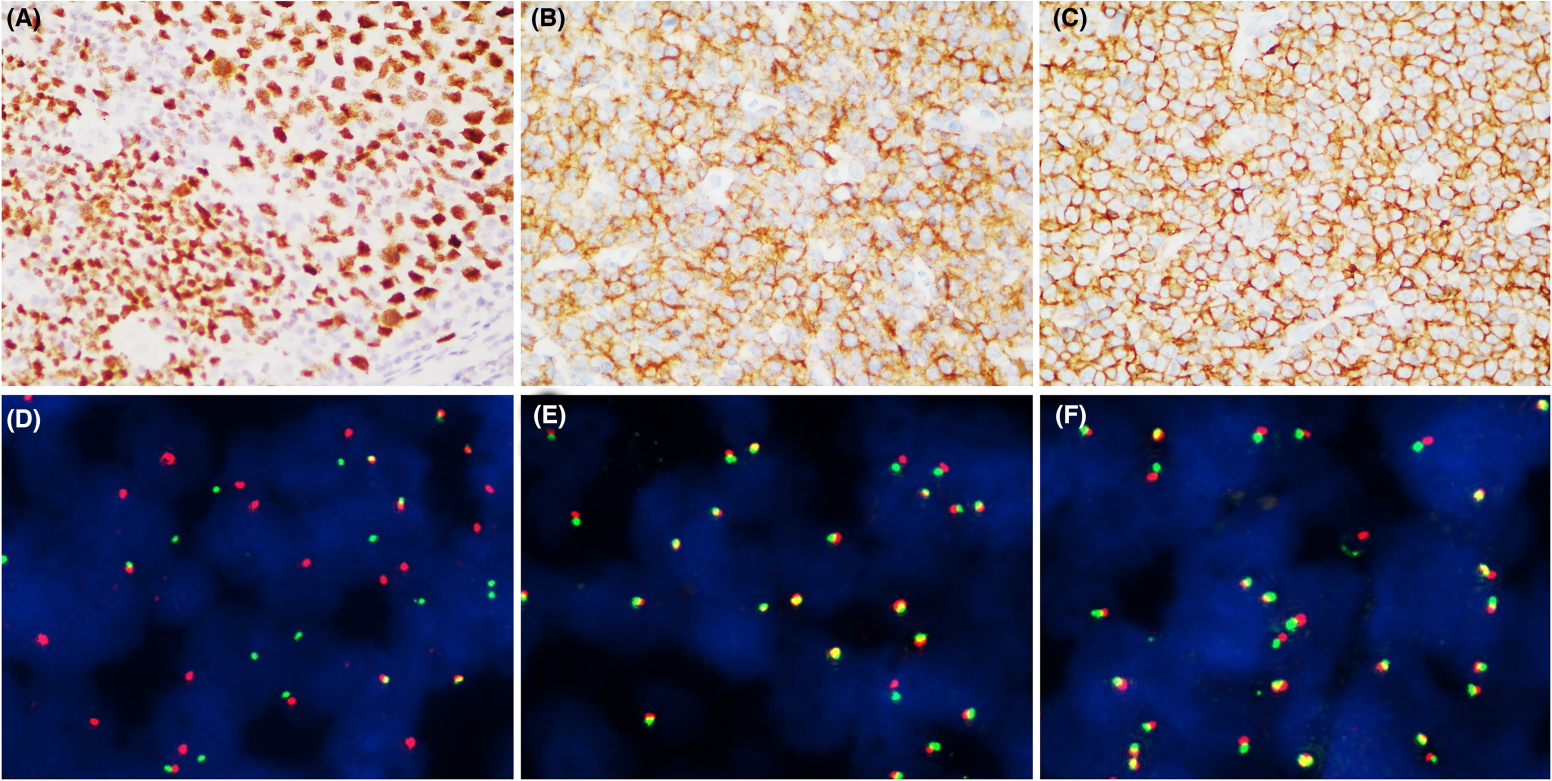
Immunohistochemically, large B‐cell lymphoma (LBCL), *IRF4*+ cases usually feature a high‐level expression of IRF4/MUM1 (A). The tumor cells often express CD10 as well (B). Some cases feature a CD5‐positive phenotype (C). Fluorescence in situ hybridization (FISH) with *IRF4* break‐apart probe shows split signals (red and green dots), suggestive of the rearrangement of the gene (D), but no rearrangement of *MYC* (E) or *BCL2* (F) is observed.

All cases stained positively for BCL6, whereas CD10 and IRF4/MUM1 positivity was detected in 60 and 56 cases, respectively. For the *IRF4*+ cohort, the staining intensity for IRF4/MUM1 was principally strong (Figure 2A), although a heterogeneous staining pattern was also demonstrated in some cases. With regard to the cell‐of‐origin subtyping, most LBCL, *IRF4+* cases (95.2%) featured a GCB subtype (Figure 2B), whereas the *IRF4*− ones more frequently (40.5%) displayed a non‐GCB phenotype (*p* = 0.001). Of note, 23.8% of *IRF4*+ tumors were CD5‐positive (Figure 2C), and the incidence was much higher than that of *IRF4*− tumors (1.3%) (*p* = 0.0010). In general, CD5‐positive LBCL, *IRF4*+ cases demonstrated similar clinicopathologic characteristics with CD5‐negative LBCL, *IRF4*− ones, although the former tended to be more frequently associated with younger patients, Waldeyer ring lesions, and medium‐sized blastoid cytology (*p* < 0.0001) (Table S1). In addition, one case each of the *IRF4*+ tumors were diffusely positive for CD23 and CD30 staining, respectively. High‐level expression of BCL2 or MYC was detected in nearly one‐third of *IRF4*+ cases each; all of those cases, however, lacked *BCL2* or *MYC* gene rearrangements (Figure 2D–F). Neither *IRF4*+ nor *IRF4*− cases were EBER‐positive.

### 3.2 Treatment and outcome

The patients received diverse treatments, including surgical excision, immunochemotherapy, with regimens most commonly of R‐CHOP (rituximab, cyclophosphamide, hydroxydaunomycin, oncovin, and prednisone) and occasionally of R‐CVP (rituximab, cyclophosphamide, vincristine, and prednisone), excisional surgery followed by chemotherapy, or sometimes combined chemotherapy and radiotherapy with or without a prior tumor mass excision. Involved site radiation therapy was administered to 10 patients in 1 month after the last cycle of chemotherapy, with a prescribed dose of 30 Gy (Table 1). While the vast majority of LBCL, *IRF4*− cases (94.9%) received chemotherapy or combined treatment, a remarkable proportion (38%) of LBCL, *IRF4*+ cases received surgical excision only and were not submitted for further chemoradiotherapy (*p* < 0.0001).

As for the outcome to the treatment (Table 2), altogether 91 patients (91%) achieved complete remission (CR), with the remaining nine LBCL, *IRF4*− patients who did not achieve CR. All of the LBCL, *IRF4*+ patients achieved CR with a significantly higher CR rate (100%) than the LBCL, *IRF4*− ones (88.6%) (*p* = 0.0030). In addition, more advanced stage and a non‐GCB phenotype also had an adverse impact on the response rate. In the logistic regression, the only variable showing an independent adverse influence on the CR rate was a higher stage (II), with a risk ratio (RR) of 6.9% and 95% confidence interval (CI) of 1.2–43.9 (*p* = 0.047).

**TABLE 2:** Univariate analysis of clinicopathologic parameters that may influence therapeutic response and survival.

	Complete remission		Overall survival		Progression‐free survival	
	*n* (%)	*p* value	5‐year probability, *n* (%)	*p* value	5‐year probability, *n* (%)	*p* value
*IRF4*−	70 (88.6)	0.0030	65 (82.3)	< 0.0001	63 (79.7)	< 0.0001
*IRF4+*	21 (100)		21 (100)		21 (100)	
Lymph nodes	54 (90.0)	0.8060	50 (83.3)	0.3083	50 (83.3)	> 0.9999
Waldeyer ring−/+nodes	35 (92.1)		34 (89.4)		32 (84.2)	
Stage I	79 (96.3)	< 0.0010	76 (92.7)	< 0.0010	75 (91.5)	< 0.0010
Stage II	12 (66.7)		10 (55.6)		9 (50.0)	
Follicular and diffuse	16 (88.9)	0.6455	15 (83.3)	0.6965	14 (77.8)	0.5591
Purely diffuse	75 (91.5)		71 (86.6)		68 (82.9)	
GCB subtype	63 (94.0)	0.0400	61 (91.0)	0.1291	59 (88.1)	0.0075
Non‐GCB subtype	28 (84.8)		25 (75.7)		25 (72.7)	
CD5−	86 (91.5)	0.1400	81 (86.2)	0.6965	79 (84.0)	> 0.9999
CD5+	5 (83.3)		5 (83.3)		5 (83.3)	

**FIGURE 3:**
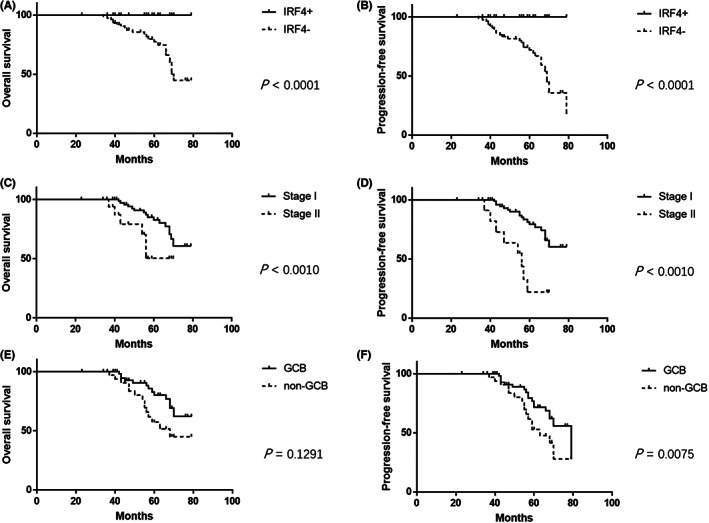
Univariate analysis demonstrates *IRF4* gene status (A, B), clinical stage (C, D), and cell‐of‐origin subtype (E, F) may have prognostic impact on the survival of young patients with primary head and neck large B‐cell lymphoma (LBCL).

After a median follow‐up of 60.5 months, the 5‐year OS and PFS in the global series were 86% and 84%, respectively. *IRF4+* patients had a better OS (100% vs. 82.3%) (*p* < 0.0001) and PFS (100% vs. 79.7%) (*p* < 0.0001) than *IRF4*− patients (Figure 3A,B). Univariate analysis also showed a more advanced stage (II) had an adverse prognostic impact on OS (55.6% vs. 92.7%) (*p* < 0.0010) (Figure 3C) and PFS (50.0% vs. 91.5%) (*p* < 0.0010) (Figure 3D), whereas the non‐GCB phenotype only correlated with a poorer PFS (*p* = 0. 0075) instead of OS (Figure 3E,F). There was no statistical difference with respect to response and survival in relation to anatomic site of involvement, follicular growth pattern, or expression status of CD5. In the multivariate analysis, *IRF4+* turned out to be the only parameter that could predict a better OS (RR, 3.7; 95% CI, 1.0–13.4) (*p* = 0.0499) and PFS (RR, 3.6; 95% CI, 1.1–11.9) (*p* = 0.0328), whereas a more advanced stage was only associated with an unfavorable PFS (RR, 7.2; 95% CI, 1.4–37.8) (*p* < 0.0001).

## 4 DISCUSSION

DLBCL represents a large group of biologically heterogeneous neoplasms, among which have been recognized some specific entities or subtypes with characteristic clinicopathologic and molecular features, such as plasmablastic lymphoma, EBV‐positive DLBCL and so on, whereas the remaining ones are provisionally labeled as DLBCL, not otherwise specified (NOS), in the WHO classification. DLBCL may affect any site of the body, including the head and neck region. Most head and neck DLBCL cases actually represent systemic disease with head and neck involvement, while about 40% of DLBCL cases are localized to head and neck sites, that is, primary head and neck lymphomas, with both nodal and extranodal involvement. Any extranodal site may be involved, including Waldeyer ring/palatine tonsil, palate, mandible, maxilla, and tongue.^8^, ^9^, ^10^ It has been recognized that the prognosis of primary head and neck DLBCL is mainly related to the subtypes, clinical stage, and International Prognostic Index, instead of sites of involvement.^11^ Specific DLBCL subtypes, such as high grade B‐cell lymphoma, usually feature their distinctive morphologic, immunophenotypic, and molecular genetic features, and are often associated with specific therapeutic strategies.

LBCL, *IRF4*+ is a rare disease affecting most frequently children and young adults, with a decreasing relative incidence with age among all LBCLs.^1^, ^5^, ^12^ It typically involves the head and neck region such as Waldeyer ring or cervical lymph nodes, and consistently expresses IRF4/MUM1 due to an *IRF4:IG* juxtaposition. Although *IRF4* rearrangement is a defining feature of this special type of lymphoma with relatively favorable prognosis, it should be noted such a molecular alteration is not specific for the entity. Since it has been found that *IRF4* rearrangement can occur in some other aggressive B‐cell lymphomas as well, especially those which are associated with *MYC* or *BCL2* rearrangement, mainly in adults.^5^, ^13^ We thus selected a large cohort of DLBCL cases occurring in youth with relatively early‐stage disease confined to the head and neck region, to compare the clinicopathologic features of LBCL, *IRF4*+ and *IRF4*− ones, and to observe the prognostic implication of *IRF4* rearrangement in these patients. The LBCL, *IRF4*+ cases in the current study present most frequently with cervical lymphadenopathy or tonsillar masses. A slight male predominance, although not statistically significant, is noticed in the *IRF4*+ patients compared with *IRF4*− ones. Histologically, the presence of a follicular growth pattern may be highly suggestive of a potential diagnosis of LBCL, *IRF4*+, which is much less frequently observed in *IRF4*− cases. The cytologic features of *IRF4*+ tumors differ from those of *IRF4−* ones, too, since the characteristic medium‐sized blastoid cells are rather commonly seen in *IRF4*+ tumors, but are generally lacking in the latter ones. In addition, the cell‐of‐origin subtype of the tumors seems critical for the diagnosis of LBCL, *IRF4*+, as all but one case feature a GCB phenotype. In contrast, 40% of *IRF4−* cases are of non‐GCB subtype. None of the tested cases in our series carries chromosomal translocations involving the *MYC* or *BCL2* gene, which is also in consistent with that documented in the literature.^1^, ^2^, ^3^, ^4^, ^5^ LBCL, *IRF4*+, therefore, seems to harbor low genomic complexity. It is noteworthy that a small fraction of LBCL, *IRF4*+ cases may also harbor *BCL6* rearrangement, most of which are young adult patients with Waldeyer ring, cervical lymph node, or bowel involvement.^1^, ^5^, ^13^, ^14^, ^15^ On the other hand, some LBCL cases with concurrent *IRF4* and *BCL2* rearrangement have been recognized as well, which frequently affects other sites of older adults, and is often morphologically atypical, and may thus represent DLBCL, NOS, rather than typical LBCL, *IRF4*+.^13^

The differential diagnosis of LBCL, *IRF4*+ includes first pediatric‐type follicular lymphoma (PTFL), which also affects children and adolescence predominantly, and present similarly with a follicular lymphoid proliferation composed of large neoplastic cells. PTFLs, however, are principally nodal lesions and rarely involve extranodal sites.^16^, ^17^, ^18^ Histologically, PTFL features purely follicular architecture without diffuse infiltration. High‐level expression of IRF4/MUM1 is rarely seen. Most importantly, PTFL uniformly lacks a *IRF4* translocation.^19^, ^20^, ^21^ It has been recently reported that LBCL, *IRF4*+ frequently harbors mutations of NF‐*k*B‐related genes such as *CARD11*. Intriguingly, *CARD11* alterations are present exclusively in cases with a diffuse growth pattern, while the *MAP2K1* gene mutations are predominantly present in PTFL,^22^ suggesting that the molecular profiles may potentially impact the morphological changes of these tumors. Conventional follicular lymphoma, Grade 3B (FL3B), or follicular LBCL, needs to be distinguished from LBCL, *IRF4*+, too, due to the similar morphological findings and a frequent IRF4/MUM1‐positive phenotype, but conventional FL3B occurs more commonly in adults, and lacks the *IRF4* translocation.^23^ Given the fact that LBCL, *IRF4*+ may express CD5, distinction with CD5‐positive DLBCL should be also taken into consideration; the later more frequently affects adults, and usually lacks follicular components and *IRF4* rearrangement.^24^ Exceptional LBCL, *IRF4*+ cases with CD5 or CD23 expression need to be distinguished from prolymphocytic progression or large cell transformation of chronic lymphocytic leukemia/small lymphocytic lymphoma. The latter, however, is basically a disease affecting elderly population, and LEF1 positivity and lack of *IRF4* rearrangement may aid in differential diagnosis as well.^25^

It has been indicated that LBCL, *IRF4*+ is a peculiar lymphoma type associated with relatively favorable prognosis.^3^ However, it remains unclear whether the better outcome of the disease is related to the genetic changes, that is, *IRF4* rearrangement, or other clinical and pathologic features of this tumor, such as the young age of the patients, relatively early‐stage disease at presentation, and a predominant GCB‐derived tumor phenotype, which are all well‐known factors associated with a better prognosis. We hence design the current study to compare the clinicopathologic characteristics of LBCL, *IRF4*+ and LBCL, *IRF4−* that occur in the head and neck region of young patients with limited stage disease. The results indicate that *IRF4+* cases feature better initial response to the treatment and better survival during follow‐up. To ascertain whether the cell‐of‐origin, that is, GCB and non‐GCB subtypes, will influence the biological behavior, we further conducted subgroup analysis (Table S2), and found that, even within GCB‐subtype cases, *IRF4* rearrangement retains to be associated with a better outcome. Further multivariate analysis has demonstrated that both clinical stage and *IRF4* status are independent prognostic factors, that is, either the presence of *IRF4* rearrangement or an earlier stage (Stage I) correlates with a relatively favorable outcome in those DLBCLs arising from the head and neck region. Of note, while Stage I correlates with a better PFS only, the rearrangement of *IRF4* correlates not only with a superior PFS, but also a better OS. Therefore, it seems reasonable and necessary to enroll both clinical staging and the detection for *IFR4* rearrangement into the prognosis‐estimating system for those young patients with limited stage disease of DLBCL involving the head and neck region. Twenty‐five percent of *IRF4*+ cases in our series were CD5‐positive LBCLs; such an incidence is almost the same as that documented in the literature. CD5 can be expressed in 5%–10% of DLBCL, NOS, and CD5‐positivity is believed to be associated with an inferior outcome of this tumor.^26^ However, in our LBCL, *IRF4*+ case series, CD5‐positivity seems to have no effects on the behavior of this tumor.

As documented in the literature, most of the LBCL, *IRF4*+ patients have been administered systemic treatment, including chemotherapy, with or without radiation, and the cure rate is usually high.^1^, ^5^, ^27^ However, there is limited existing evidence to support treatment de‐escalation.^27^, ^28^, ^29^, ^30^ For example, whether those patients with lymphoma lesions completely removed by surgery still need complementary systemic treatment remains unclear. Nearly 40% of all *IRF4*+ patients in our series received mere surgical resection, without additional chemotherapy or radiation, all of whom turned to be cured later. These patients were all characterized by localized lesions (Stage I and no bulky disease), and other clinicopathologic characteristics comparable with those patients who had received systemic treatment (Table S3). Based on these findings, a more conservative therapeutic strategy of complete resection followed by watchful wait might be adequate as well as safe for those with solitary, resectable lesions, which may certainly aid in avoiding possible overtreatment.

In conclusion, rearrangement of the *IRF4* gene not only defines a group of LBCL with distinct clinicopathologic features, but also is of practical implication for prognosis prediction and therapeutic strategy decision. We have compared for the first time the pathologic and prognostic differences between *IRF4*+ and *IRF4−* primary head and neck DLBCLs, and found that *IRF4* rearrangement may represent an independent prognostic factor indicative of better outcome. These findings further unravel the heterogeneity of DLBCL arising in young patients, which may provide additional parameters for risk stratification and individualized treatment.

## AUTHOR CONTRIBUTIONS

**Xiang‐Nan Jiang:** Data curation (lead); formal analysis (lead); funding acquisition (equal); investigation (equal); methodology (lead); project administration (equal); software (lead); visualization (lead); writing – original draft (lead); writing – review and editing (equal). **Fang Yu:** Data curation (equal); investigation (equal); resources (equal). **Tian Xue:** Data curation (equal); investigation (equal); methodology (equal). **Qing‐Xin Xia:** Resources (equal). **Qian‐Ming Bai:** Investigation (equal); methodology (equal); software (equal). **Bao‐Hua Yu:** Resources (equal). **Ruo‐Hong Shui:** Resources (equal). **Xiao‐Yan Zhou:** Methodology (equal); validation (equal). **Xiong‐Zeng Zhu:** Validation (equal). **Jun‐Ning Cao:** Validation (equal). **Xiao‐Nan Hong:** Validation (equal). **Xiao‐Qiu Li:** Conceptualization (lead); investigation (lead); project administration (lead); resources (lead); funding acquisition (equal); supervision (lead); validation (lead); writing – review and editing (equal).

## FUNDING INFORMATION

This work was supported by grants from the Chinese Society of Clinical Oncology (No. Y‐XD202001‐0152), Science and Technology Commission of Shanghai Municipality (No. 22YF1408200), and Shanghai Anticancer Association EYAS Project (No. SHCY‐ZH‐202106).

## CONFLICT OF INTEREST STATEMENT

The authors declare that the research was conducted in the absence of any commercial or financial relationships that could be construed as a potential conflict of interest.

## Supporting information


Figure S1.

Figure S2.

Table S1.

Table S2.

Table S3.

## Data Availability

I confirm that my article contains a Data Availability Statement even if no data is available (list of sample statements) unless my article type does not require one.I confirm that I have included a citation for available data in my references section, unless my article type is exempt.
