# Supplementary figures and images for: IRF4 rearrangement may predict favorable prognosis in children and young adults with primary head and neck large B‐cell lymphoma
Source: Cancer Med. 2023 Apr 20;12(9):10684–93. doi: 10.1002/cam4.5828 (PMC10225228; doi:10.1002/cam4.5828)

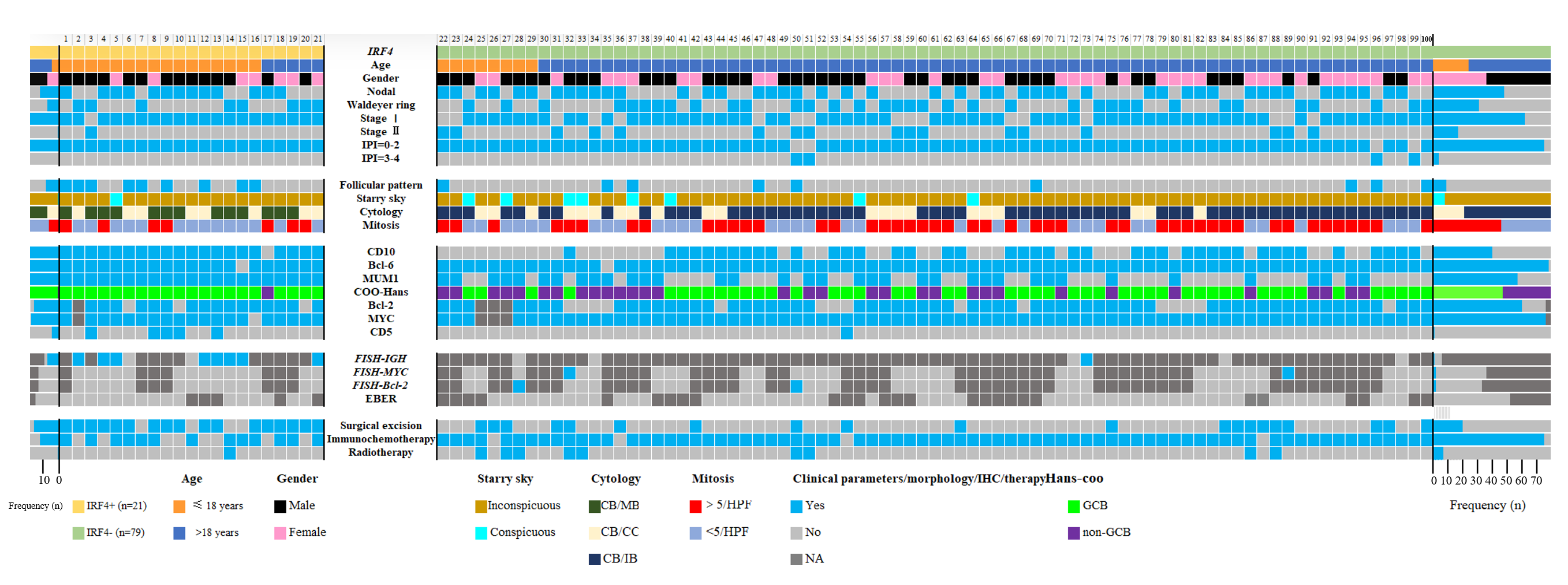

Supplement: Supplementary file 1 — Figure S1. [file CAM4-12-10684-s001.tif]

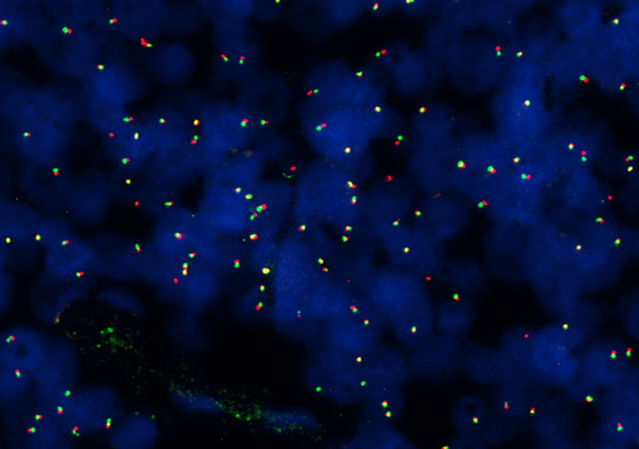

Supplement: Supplementary file 2 — Figure S2. [file CAM4-12-10684-s002.tif]
