# Supplementary material for: IRF4 rearrangement may predict favorable prognosis in children and young adults with primary head and neck large B‐cell lymphoma
Source: Cancer Med. 2023 Apr 20;12(9):10684–93. doi: 10.1002/cam4.5828 (PMC10225228; doi:10.1002/cam4.5828)
Supplement: Supplementary file 3 — Table S1. [file CAM4-12-10684-s003.docx]

**Supplementary Table 1. Comparison of CD5-positive LBCL, *IRF4*+ cases with CD5-negative LBCL, *IRF4*+ cases**

|  | **CD5- (n=16)** | **CD5+ (n=5)** | ***P* value** |
| --- | --- | --- | --- |
| ≤ 18 yrs, n (%)  > 18 yrs, *n* (%) | 11 (68.8)  5 (31.2) | 5 (100)  0 (0) | < 0.0001 |
| Male  Female | 10 (62.5)  6 (37.5) | 4 (80.0)  1 (20.0) | 0.0118 |
| Lymph nodes, n (%)  Waldeyer ring, n (%) | 11 (68.8)  5 (31.2) | 2 (40.0)  3 (60.0) | < 0.0001 |
|  |  |  |  |
| Stage I  Stage II | 15 (93.8)  1 (6.2) | 5 (100)  0 (0) | 0.0289 |
| IPI score 0-2, *n* (%)  IPI score 3-4, *n* (%) | 16 (100)  0 (0) | 5 (100)  0 (0) | > 0.9999 |
| Follicular and diffuse, *n* (%)  Purely diffuse, *n* (%) | 7 (43.8)  9 (56.2) | 2 (40.0)  3 (60.0) | 0.6675 |
| CB/CC, *n* (%) | 8 (50.0) | 0 (0) | < 0.0001 |
| CB/MB, *n* (%) | 8 (50.0) | 5 (100) |  |
|  |  |  |  |
| GCB subtype, *n* (%)  Non-GCB subtype, *n* (%) | 15 (93.8)  1 (6.2) | 5 (100)  0 (0) | 0.0289 |
