# Supplementary material for: IRF4 rearrangement may predict favorable prognosis in children and young adults with primary head and neck large B‐cell lymphoma
Source: Cancer Med. 2023 Apr 20;12(9):10684–93. doi: 10.1002/cam4.5828 (PMC10225228; doi:10.1002/cam4.5828)
Supplement: Supplementary file 4 — Table S2. [file CAM4-12-10684-s005.docx]

**Supplementary Table 2. Subgroup analysis** **of clinicopathologic parameters that may influence therapeutic response and survival**

|  | **Complete remission** | | | | **Overall survival** | | | **Progression-free survival** | | |
| --- | --- | --- | --- | --- | --- | --- | --- | --- | --- | --- |
|  | *n* (%) | | *P* value | 5-year probability *n* (%) | | | *P* value | | 5-year probability *n* (%) | *P* value |
| *IRF4-*, follicular and diffuse  *IRF4+*, follicular and diffuse | 8 (88.9)  9 (100) | 0.0004 | | | 8 (88.9)  9 (100) | 0.0004 | | 8 (88.9)  9 (100) | | 0.0004 |
| *IRF4-*, purely diffuse  *IRF4+*, purely diffuse | 60 (89.6)  12 (100) | 0.0002 | | | 55 (82.1)  12 (100) | < 0.0001 | | 53 (79.1)  12 (100) | | < 0.0001 |
| *IRF4-*, GCB  *IRF4+*, GCB  *IRF4-*, non-GCB  *IRF4+*, non-GCB | 42 (89.4)  20 (100)  28 (87.5)  1 (100) | 0.0007  0.0003 | | | 44 (93.6)  20 (100)  21 (65.6)  1 (100) | 0.0041  < 0.0001 | | 43 (91.5)  20 (100)  20 (62.5)  1 (100) | | 0.0028  < 0.0001 |
| *IRF4-*, CD5+  *IRF4+*, CD5+  *IRF4-*, CD5-  *IRF4+*, CD5- | 1 (100)  5 (100)  69 (88.5)  16 (100) | > 0.9999  0.0007 | | | 1 (100)  5 (100)  64 (82.1)  16 (100) | > 0.9999  < 0.0001 | | 1 (100)  5 (100)  62 (79.5)  16 (100) | | > 0.9999  < 0.0001 |
