# Supplementary material for: IRF4 rearrangement may predict favorable prognosis in children and young adults with primary head and neck large B‐cell lymphoma
Source: Cancer Med. 2023 Apr 20;12(9):10684–93. doi: 10.1002/cam4.5828 (PMC10225228; doi:10.1002/cam4.5828)
Supplement: Supplementary file 5 — Table S3. [file CAM4-12-10684-s004.docx]

**Supplementary Table 3. Comparison of the 8 LBCL, *IRF4*+ patients who received resection only with all the LBCL, *IRF4*+ patients**

|  | ***All* (n=21)** | **Resection only (n=8)** | ***P* value** |
| --- | --- | --- | --- |
| ≤ 18 yrs, n (%)  > 18 yrs, *n* (%) | 16 (76.2)  5 (23.8) | 6 (75.0)  2 (25.0) | > 0.9999 |
| Male  Female | 14 (66.7)  7 (33.3) | 8 (100)  0 (0) | < 0.0001 |
| Stage I  Stage II | 20 (95.2)  1 (4.8) | 8 (100)  0 (0) | 0.0594 |
| IPI score 0-2, *n* (%)  IPI score 3-4, *n* (%) | 21 (100)  0 (0) | 8 (100)  0 (0) | > 0.9999 |
| Follicular and diffuse, *n* (%)  Purely diffuse, *n* (%) | 9 (42.9)  12 (57.1) | 4 (50.0)  4 (50.0) | 0.3207 |
| GCB subtype, *n* (%)  Non-GCB subtype, *n* (%) | 20 (95.2)  1 (4.8) | 7 (87.5)  1 (12.5) | 0.1262 |
| Lymph node, *n* (%)  Waldeyer ring, *n* (%) | 13 (61.9)  8 (38.1) | 5 (61.9)  3 (38.1) | > 0.9999 |
| CD5+, *n* (%)  CD5-, *n* (%) | 5 (23.8)  16 (76.2) | 4 (50%)  4 (50%) | 00.0001 |
